# The effect of apple cider vinegar on lipid profiles and glycemic parameters: a systematic review and meta-analysis of randomized clinical trials

**DOI:** 10.1186/s12906-021-03351-w

**Published:** 2021-06-29

**Authors:** Amir Hadi, Makan Pourmasoumi, Ameneh Najafgholizadeh, Cain C. T. Clark, Ahmad Esmaillzadeh

**Affiliations:** 1 Department of Clinical Nutrition, School of Nutrition and Food Science, Isfahan University of Medical Sciences, Isfahan, Iran; 2 Gastrointestinal & Liver Diseases Research Center (GLDRC), Guilan University of Medical Sciences (GUMS), Rasht, Iran; 3 Department of Microbiology, Naein Branch, Islamic Azad University Isfahan, Isfahan, Iran; 4 Centre for Intelligent Healthcare, Coventry University, Coventry, CV1 5RW UK; 5 Department of Community Nutrition, School of Nutritional Sciences and Dietetics, Tehran University of Medical Sciences, P. O. Box: 14155-6117, Tehran, Iran; 6 Obesity and Eating Habits Research Center, Endocrinology and Metabolism Molecular-Cellular Sciences Institute, Tehran University of Medical Sciences, Tehran, Iran; 7 Department of Community Nutrition, School of Nutrition and Food Science, Food Security Research Center, Isfahan University of Medical Sciences, Esfahan, Iran

**Keywords:** Apple cider vinegar, Lipid profiles, Clinical trials, Meta-analysis, Glycemic indices

## Abstract

**Background:**

Elevated lipid profiles and impaired glucose homeostasis are risk factors for several cardiovascular diseases (CVDs), which, subsequently, represent a leading cause of early mortality, worldwide. The aim of the current study was to conduct a systematic review and meta-analysis of the effect of apple cider vinegar (ACV) on lipid profiles and glycemic parameters in adults.

**Methods:**

A systematic search was conducted in electronic databases, including Medline, Scopus, Cochrane Library, and Web of Knowledge, from database inception to January 2020. All clinical trials which investigated the effect of ACV on lipid profiles and glycemic indicators were included. Studies were excluded if ACV was used in combination with other interventions or when the duration of intervention was < 2 weeks. To account for between-study heterogeneity, we performed meta-analysis using a random-effects model.

**Results:**

Overall, nine studies, including 10 study arms, were included in this meta-analysis. We found that ACV consumption significantly decreased serum total cholesterol (− 6.06 mg/dL; 95% CI: − 10.95, − 1.17; *I*^*2*^: 39%), fasting plasma glucose (− 7.97 mg/dL; 95% CI: − 13.74, − 2.21; *I*^*2*^: 75%), and HbA1C concentrations (− 0.50; 95% CI: − 0.90, − 0.09; *I*^*2*^: 91%). No significant effect of ACV consumption was found on serum LDL-C, HDL-C, fasting insulin concentrations, or HOMA-IR. The stratified analysis revealed a significant reduction of serum TC and TG in a subgroup of patients with type 2 diabetes, those who took ≤15 mL/day of ACV, and those who consumed ACV for > 8-weeks, respectively. Furthermore, ACV consumption significantly decreased FPG levels in a subgroup of studies that administered ACV for > 8-weeks. Further, ACV intake appeared to elicit an increase in FPG and HDL-C concentrations in apparently healthy participants.

**Conclusion:**

We found a significant favorable effect of ACV consumption on FPG and blood lipid levels.

**Supplementary Information:**

The online version contains supplementary material available at 10.1186/s12906-021-03351-w.

## Background

Cardiovascular diseases (CVDs), collectively, are regarded as the number one cause of early mortality, worldwide [30]. According to the World Health Organization (WHO), approximately 17.7 million deaths were attributable to CVD in 2015, and it is projected to account for more than 23.6 million by 2030 [2]. Indeed, given the high economic costs of CVDs on healthcare systems, especially in developing countries, prevention and management of CVDs is of high priority.

Dyslipidemia, in particular hypercholesterolemia, and hyperglycemia are regarded as the most important contributors to CVD events [24, 25]. Lifestyle modifications (dietary changes and physical activity), along with pharmacological interventions, such as statins, fibrates, and insulin sensitizers, are routinely used to manage these metabolic disorders [1, 14, 19, 26]. However, low adherence to lifestyle recommendations and reported adverse reactions of synthetic agents [38, 41] highlights the necessity of discerning novel and efficacious approaches. In this line, the benefical effect of nutraceutics and fuctional foods on human health have been well-documented [8, 36]. In contemporary research and practice, plants and their derivatives have attracted a lot of interest for their beneficial effects in controlling lipid profile and glycemic status [5, 15, 37]. Indeed, one of the most popular plant derivatives in this regard is vinegar.

Apple cider vinegar (ACV) is one of the three most common types of vinegar, produced by fermenting apples [3]. This acidic solution is consumed throughout the world as a flavoring and preservative agent in foods [22]. ACV contains a variety of flavonoids, such as gallic acid, catechin, caffeic acid, and ferulic acid [11, 31]. Animal experiments have reported that ACV has a variety of pharmacological functions, including anti-oxidant, anti-inflammatory, anti-diabetic, anti-hypertensive, and anti-hyperlipidemic properties [7, 18, 32, 39]. The effects of ACV on serum lipid parameters and glycemic markers have been investigated in several randomized clinical trials [4, 12, 16, 22, 23, 27, 33]; however, the results are equivocal. Indeed, some investigations have reported beneficial effects following ACV consumption on the aforementioned parameters [16, 22, 23, 27], although others failed to detect any effects [4, 12, 33]. It is conceivable that such contradictory findings might be due to the differences in study design and/or characteristics of participants (age, sex, clinical condition).

To the best of our knowledge, there has been no systematic compilation of the previously reported effects of ACV on lipid profiles and glycemic status. Therefore, in the current study, we performed a systematic review and meta-analysis of all published clinical trials to provide a more precise estimation of the effects of ACV on serum lipid parameters and glycemic markers in adults.

## Methods

The present systematic review and meta-analysis was planned, conducted, and reported according to the guidelines of the 2009 Preferred Reporting Items for Systematic Reviews and Meta- Analysis (PRISMA) statement [29].

### Search strategy

A comprehensive literature search was performed to identify and appraise investigations that had assessed the effects of ACV supplementation on lipid profiles and glycemic parameters. Electronic databases, including Medline, Scopus, Cochrane Library, and Web of Knowledge, were searched from database inception to January 2020. The relevant keywords were used in combination with the Medical Subject Heading (MeSH) terms, search tag, and boolean operators (AND, OR, NOT) (Supplemental Table 1). The search keywords were seldom based on vinegar and related phrases to minimize the chance of missing studies which reported lipids or glycemic related markers as secondary outcomes. Additionally, the reference lists of related review articles and the retrieved studies were also hand-searched to detect eligible trials that might have been missed.

### Study selection

After excluding duplicate studies, two authors (AH and MP) independently reviewed articles based on titles, abstracts, or full-texts to identify relevant studies. Eventually, original studies were included in the present meta-analysis if they: 1) were randomized clinical trials; 2) administered ACV as the intervention; 3) enrolled adult participants (aged ≥18 years); 4) reported lipid and glycemic parameters as the outcomes of interest. Studies that met the following criteria were excluded: 1) ACV was used in combination with other interventions; 2) studies with an intervention duration of fewer than 2 weeks; 3) studies that did not report relevant effect sizes. Table 1 shows the PICOS (participants, intervention/exposure, comparisons, outcomes, and study design) criteria which was used to define the research question.

**Table 1:** PICO (participants, intervention/exposure, comparison, outcomes, and study design) criteria for inclusion and exclusion of studies

Parameters	Descriptions
Participants	Adult
Intervention	Apple cider vinegar supplementation
Comparison	Any comparator/control that incorporated a nonintervention group
Outcomes	lipid profile levels and glycemic indices
Setting	Randomized controlled trials

### Data extraction and risk of bias assessment

The main information from eligible studies were extracted by two researchers independently and the following data were abstracted: first author’s last name, years of publication, study location, sample size, participant’s health condition, mean age of subjects, design and duration of intervention, comparison groups, type of intervention, and main outcome. Any disagreement was settled by face-to-face discussion. Furthermore, in instances of unclear information in included studies, clarification was soughtby emailing the relevant studies’ corresponding authors.

The Cochrane Risk of Bias Tool was applied to assess potential risks of bias in included RCTs [17]. This scale is based on several items to assess the adequacy of random sequence generation, allocation concealment, blinding as well as detection of incomplete outcome data, selective outcome reporting, and other potential sources of bias. Based on recommendations of the Cochrane Handbook, judgment of each item appears by “Low”, “High”, and “Unclear” risk of bias.

### Statistical analysis

All statistical analyses were conducted using the Cochrane Program Review Manager Version 5.3 (The Cochrane Collaboration, 2011, The Nordic Cochrane Centre, Copenhagen) and STATA version 11 software. To estimate the overall effect size, the mean differences (MD) and standard deviations (SDs) of all outcomes of interest, including fasting plasma glucose (FPG), fasting insulin, HbA1C, homeostasis model assessment of insulin resistance (HOMA-IR), triacylglycerol (TG), total cholesterol (TC), low-density lipoprotein cholesterol (LDL-C), and high-density protein cholesterol (HDL-C), were collected. Net changes in these variables in intervention and control groups were computed by the subtraction of the post-measurement data from pre-intervention values if they were not already available in the original study. As SD of mean change was provided in only 2 studies [4, 22], we calculated missing SDs of change for other studies according to the following formula: [SD = square root [(SD pre-treatment)^2^ + (SD post-treatment)^2^ - (2 r × SD pre-treatment × SD post-treatment)] [17]; where the correlation coefficient (r) was examined using the following equation: [r = (SD^2^_Baseline_ + SD^2^_Final_-SD_Change_) / (2× SD _Baseline_ ×SD _Final_)] [17], in which required data were obtained from the study of Bashiri et al. [4]. Based on this, r for TG was considered as 0.73, for TC as 0.76, for LDL-C as 0.68, and for HDL-C as 0.66. The corresponding r for FPG, HbA1C, HOMA-IR and fasting insulin was assumed to be 0.66 for all [17]. To account for probable between-study heterogeneity, we applied a random-effects model in our analyses to estimate the overall effect size [10]. Inter-study heterogeneity was assessed by Cochran’s *Q* and *I*^*2*^ statistics, where *P* < 0.10 or *I*^*2*^ > 50% was regarded as possessing potential statistical heterogeneity (J. Higgins & Green). The source of heterogeneity was then explored through subgroup analysis. Effect sizes were presented as weighted mean differences with 95% confidence intervals (CI). The sensitivity analysis was applied to assess the influence of individual studies on the overall findings. Egger’s regression asymmetry test and Begg’s rank-correlation test were performed to explore potential publication bias [13, 40]. A random-effects meta-regression was conducted to identify the potential impact of putative moderators, including baseline measures, amount, and duration of apple vinegar administration on estimated net changes of outcome [6]. *P*-values < 0.05 were, a priori, considered as statistically significant.

## Results

**Fig. 1:**
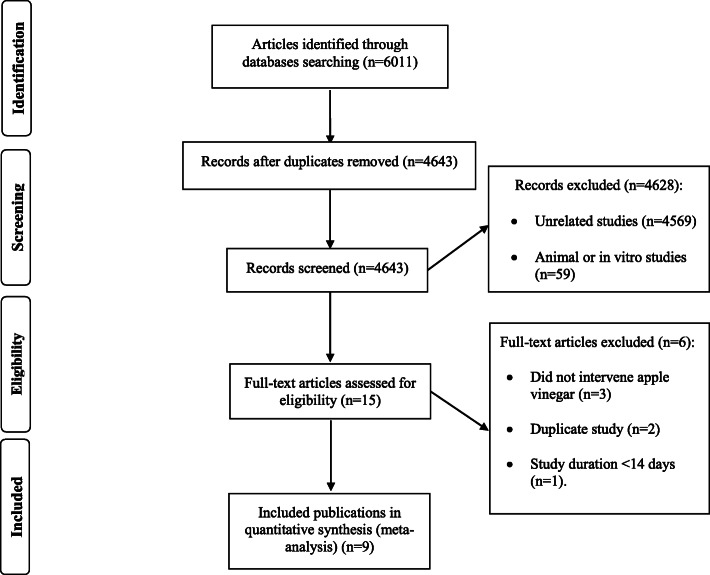
Flow chart of the process of the study selection

The study selection process, including the number and the reason for excluded studies are illustrated in Fig. 1. In brief, the primary electronic search yielded 4643 unduplicated records, where 4628 articles were removed following title/abstract screening, and thus, 15 studies were selected for further assessment. Six studies were excluded due to duplicated data (*n* = 2), duration of intervention < 1 week (*n* = 1), and the use of non-apple vinegar (*n* = 3). One selected study [23] had used 2 different doses of ACV; therefore, it was considered as 2 interdependent active arms. Therefore, 9 studies, including 10 study arms, were included in the final meta-analysis.

### Study characteristics

The main characteristics of included clinical trials are provided in Table 2. Nine studies [4, 12, 16, 20, 22, 23, 27, 28, 33], comprising 686 total participants, with a mean age of 49.5 y, met the eligibility criteria and were selected for qualitative and quantitative analysis. These studies were published between 2008 and 2019, and had been conducted in Iran [4, 12, 22, 27, 28], USA [33], Japan [23], Pakistan [20], and Tunisia [16], respectively. Aside from one study [16], which did not report the gender of participants, all included studies had recruited both genders. Participants’ clinical conditions were different across the included studies; where five trials had enrolled diabetic patients [12, 16, 20, 27, 28], 2 studies had included obese and/or overweight participants [22, 23], one study had recruited type 2 diabetic patients with dyslipidemia [4], and one study did not report the condition of subjects [33]. All trials were of parallel design and the duration of intervention ranged between 30 and 90 days. The dose of ACV varied from 15 to 770 mL/day. Participants in control groups were prescribed water/beverage [16, 20, 23, 27, 28, 33] or a restricted-calorie diet [22]. Two studies [4, 12] had no administration in control groups.

**Table 2:** Characteristics of included studies

First author (publication year)	Country	Number and gender (F/M)	Study participants health condition	Mean age	Clinical Trial design /randomized/Blinding	Duration	Comparison group	Amount of vinegar intake	Reported outcomes
Bashiri et al. (2014) [4]	Iran	Number: 62 (Both gender)	Type 2 diabetic patients with dyslipidemia	Range: 25–65 Intervention: 49.47 ± 8.02 Placebo: 52.1 ± 7.87	Parallel/ Yes/No	8 weeks	–	20 mL/day	TG TC HDL LDL
Halima et al. (2017) [16]	Tunisia	Number: 44 (NR)	Type 2 diabetes	range: 40–65 Intervention: NR Placebo: NR	Parallel/ Yes/ Yes	1 month	water	15 mL/day	FPG TG TC HDL LDL
Kondo et al. (2009) [23]	Japan	Number: 155 (Both gender)	Obese	Range:25–60 Intervention (high-dose): 43.4 ± 9.5 Intervention (low-dose): 44.7 ± 9.7 Placebo: 44.1 ± 9.6	Parallel/ Yes/ Yes	12 weeks	Beverage	15 mL/day	TG TC HDL-C LDL-C FPG HbA_1_C
								30 mL/day	
Mahmoodi et al. (2013) [27]	Iran	Number: 60 (Both gender)	Type 2 diabetic	Range:30–60	Parallel/ NR/ Yes	1 month	Water	15 mL/day	TG TC HDL-C LDL-C FPG HbA_1_C
Panetta et al. (2013) [33]	USA	Number: 97 (Both gender)	NR	Intervention: 57.7 ± 9.33 Placebo: 56.1 ± 12.58	Parallel/ Yes/ Yes	8 weeks	Balsamic vinegar solution diluted in water	30 mL/day	TG TC HDL-C LDL-C HbA_1_C
Khezri et al. (2018) [22]	Iran	Number: 44 (Both gender)	Obese and overweight	Range: 27–40 Intervention: 42.5 ± 9 Placebo: 45 ± 11	Parallel/ Yes/ No	12 weeks	Restricted calorie diet	30 mL/day plus restricted calorie diet.	TG TC HDL-C LDL-C
Ebrahimi-Mamaghani et al. (2009) [12]	Iran	Number: 38 (Both gender)	Type 2 diabetic	Intervention: 54.6 ± 13.1 Placebo: 53.8 ± 9.0	Parallel/ Yes/ No	8 weeks	–	770 mL/day	TG TC HDL-C LDL-C FPG
Mohammadpourhodki et al. (2019) [28]	Iran	Number: 76 (Both gender)	Type 2 diabetes	Range: 18–65 Intervention: 49.2 ± 4.3 Placebo: 49.2 ± 4.3	Parallel/ Yes/No	8 weeks	Water	20 mL/day	FPG HbA1C
Kausar et al. (2019) [20]	Pakistan	Number: 110 (Both gender)	Type 2 diabetes	Range: 30–60 Intervention: 51.16 ± 7.91 Placebo: 50.49 ± 7.78	Parallel/ Yes/Yes	3 month	Water with artificial flavor	15 mL/day	TG TC HDL-C LDL-C FPG HbA_1_C

*Abbreviations*: *TG* triacylglycerol, *TC* total-cholesterol, *LDL-C* Low-density lipoprotein cholesterol, *HDL-C* High-density lipoprotein cholesterol, *FPG* Fasting plasma glucose, *HbA1C* hemoglobin A1C, *NR* Not Reported

### Risk of bias assessment

The author’s judgment on each criterion of the risk of bias assessment is presented in Table 3. In summary, 8 trials [4, 12, 16, 20, 22, 23, 28, 33] were randomized, however, only 3 studies [20, 22, 33] had provided enough information regarding allocation concealment. Three studies [20, 23, 33] were blinded. Seven studies [4, 12, 20, 22, 23, 28, 33] had low attrition bias and/or described the reason of participants’ withdrawal. Three trials had reported the controlling of other factors that may influence outcomes [4, 20, 22].

**Table 3:** The summary of review authors’ judgments about each risk of bias item for included studies

Study	Random sequence generation	Allocation concealment	Blinding	Incomplete outcome data	Selective reporting	Other bias
Bashiri et al. [4]	L	U	H	L	L	L
Halima et al. [16]	**L**	**U**	**U**	**U**	**L**	**U**
Kondo et al. [23]	L	U	L	L	L	U
Mahmoodi et al. [27]	**U**	**H**	**U**	**U**	**L**	**U**
Panetta et al. [33]	L	L	L	L	L	U
Khezri et al. (2018) [22]	L	L	H	L	L	L
Ebrahimi-Mamaghani et al. [12]	**L**	**U**	**H**	**L**	**L**	**U**
Mohammadpourhodki et al. (2019) [28]	**L**	**U**	**H**	**L**	**L**	**U**
Kausar et al. (2019) [20]	L	L	L	L	L	L

*H* high risk of bias, *L* low risk of bias, *U* unclear or unrevealed risk of bias. Criteria defined for risk of bias assessment are according to the Cochrane guidelines

### Meta-analysis

#### The effect of ACV administration on lipid profile

**Fig. 2:**
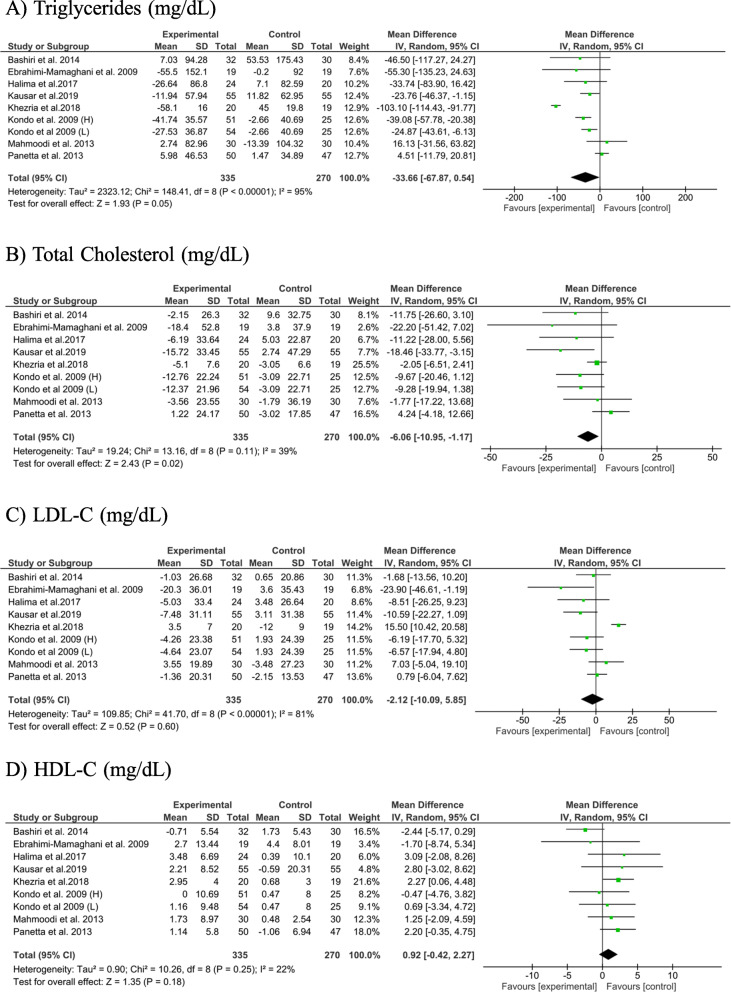
The meta-analysis results of the effect of apple cider vinegar administration on lipids profiles. Kondo et al. study administrated apple cider vinegar in 2 different dosages which showed as “L” (lower dose) and “H” (higher dose) in figure

Findings from 8 studies, with 9 effect sizes, revealed that ACV consumption significantly decreased serum TC concentrations (− 6.06 mg/dL; 95% CI: − 10.95, − 1.17, *P* = 0.02; *I*^*2*^: 39%). In addition, a trend toward a significant reduction in serum TG levels was also seen following ACV consumption (− 33.66 mg/dL; 95% CI: − 67.87, 0.54, *P* = 0.05; *I*^*2*^: 95%). No significant effect of ACV consumption on serum LDL-C (− 2.12 mg/dL; 95% CI: − 10.09, 5.85, *P* = 0.60; *I*^*2*^: 81%) and HDL-C concentrations was found after ACV consumption (0.92 mg/dL; 95% CI: − 0.42, 2.27, *P* = 0.18; *I*^*2*^: 22%) (Fig. 2).

To discern the source of heterogeneity, we performed subgroup analyses based on participant’s condition, dose of ACV consumption, and the duration of intervention. In these analyses, we found a notable decrease in both TG and TC concentrations in studies conducted on type 2 diabetic patients (TG: − 22.46 mg/dL; 95% CI: − 40.27, − 4.65; *I*^*2*^: 0%; TC: − 11.51 mg/dL; 95% CI: − 18.16, − 4.86; *I*^*2*^: 0%), as well as in studies with an ACV dose of ≤15 mL/day (TG: − 21.91 mg/dL; 95% CI: − 35.23, − 8.60; *I*^*2*^: 0%; TC: − 10.22 mg/dL; 95% CI: − 16.46, − 3.98; *I*^*2*^: 0%) and studies with > 8-weeks of intervention (TG: − 48.22 mg/dL; 95% CI: − 92.83, − 3.60; *I*^*2*^: 96%; TC: − 7.61 mg/dL; 95% CI: − 14.29, − 0.94; *I*^*2*^: 49%). However, no significant reduction in these variables was found in studies conducted on non-diabetics, studies that administered > 15 mL/day, and those with a duration of intervention of ≤8-weeks. In addition, a significant increase in serum HDL-C levels was observed in studies that recruited non-diabetics (HDL-C: 1.73 mg/dL; 95% CI: 0.28, 3.18; *I*^*2*^: 0%) (Table 4).

**Table 4:** subgroup analysis

Variables	Subgroup analysis based on		Number of trials	Mean difference (95%CI)	Within study heterogeneity	Between study heterogeneity
TG	Participants condition	Type 2 diabetes	**5**	**−22.46 (−40.27, −4.65)**	0%	0.001
		Other condition	4	−40.86 (−93.61, 11.88)	97%	
	Amount of apple vinegar	> 15 mL/day	5	− 47.59 (−101.58, 6.67)	96%	< 0.001
		≤ 15 mL/day	4	**−21.91 (−35.23, −8.60)**	0%	
	Duration	> 8 weeks	4	**−48.22 (−92.83, − 3.60)**	96%	< 0.001
		≤ 8 weeks	5	−9.51 (− 33.63, 14.60)	32%	
TC	Participants condition	Type 2 diabetes	**5**	**−11.51 (− 18.16, −4.86)**	0%	0.01
		Other condition	4	−3.21 (−8.81, 2.40)	47%	
	Amount of apple vinegar	> 15 mL/day	5	−4.00 (−10.19, 2.19)	46%	0.03
		≤ 15 mL/day	4	**−10.22 (− 16.46, −3.98)**	0%	
	Duration	> 8 weeks	4	**−7.61 (−14.29, −0.94)**	49%	0.75
		≤ 8 weeks	5	−5.71 (−14.33, 2.92)	49%	
LDL-C	Participants condition	Type 2 diabetes	5	−5.39 (− 14.31, 3.53)	48%	0.002
		Other condition	4	1.62 (−9.85, 5.85)	87%	
	Amount of apple vinegar	> 15 mL/day	5	−0.59 (−11.73, 10.55)	85%	0.003
		≤ 15 mL/day	4	−4.34 (−12.51, 3.84)	38%	
	Duration	> 8 weeks	4	−1.41 (− 16.36, 13.55)	90%	0.02
		≤ 8 weeks	5	−1.66 (−8.83, 5.51)	38%	
HDL-C	Participants condition	Type 2 diabetes	5	0.21 (−2.17, 2.60)	34%	0.10
		Other condition	4	**1.73 (0.28, 3.18)**	0%	
	Amount of apple vinegar	> 15 mL/day	5	0.39 (−1.78, 2.57)	56%	0.49
		≤ 15 mL/day	4	1.61 (−0.52, 3.75)	0%	
	Duration	> 8 weeks	4	1.62 (− 0.07, 3.30)	0%	0.32
		≤ 8 weeks	5	0.52 (−1.75, 2.79)	48%	
FPG	Participants condition	Type 2 diabetes	5	−16.28 (−33.02, 0.47)	83%	0.69
		Other condition	2	**−3.53 (−6.70, −0.37)**	0%	
	Amount of apple vinegar	> 15 mL/day	3	−16.12 (−41.31, 9.07)	87%	0.15
		≤ 15 mL/day	4	−4.10 (−8.98, 0.76)	52%	
	Duration	> 8 weeks	**3**	**−3.78 (− 6.90, −0.66)**	0%	0.55
		≤ 8 weeks	4	−17.14 (−38.15, 3.86)	86%	
HbA1C	Participants condition	Type 2 diabetes	3	−0.77 (−1.56, 0.02)	88%	0.001
		Other condition	3	−0.07 (− 0.32, 0.18)	0%	
	Amount of apple vinegar	> 15 mL/day	3	−0.60 (−1.54, 0.33)	92%	0.13
		≤ 15 mL/day	3	−0.24 (− 0.51, 0.03)	10%	
	Duration	> 8 weeks	3	−0.72 (−1.58, 0.15)	90%	0.01
		≤ 8 weeks	3	−0.14 (− 0.43, 0.15)	23%	

The effect size was obtained from random effect model. *Abbreviations*: *TG* triacylglycerol, *TC* total-cholesterol, *LDL-C* Low-density lipoprotein cholesterol, *HDL-C* High-density lipoprotein cholesterol, *FPG* Fasting Plasma Glucose

#### The effect of ACV administration on glycemic profile

**Fig. 3:**
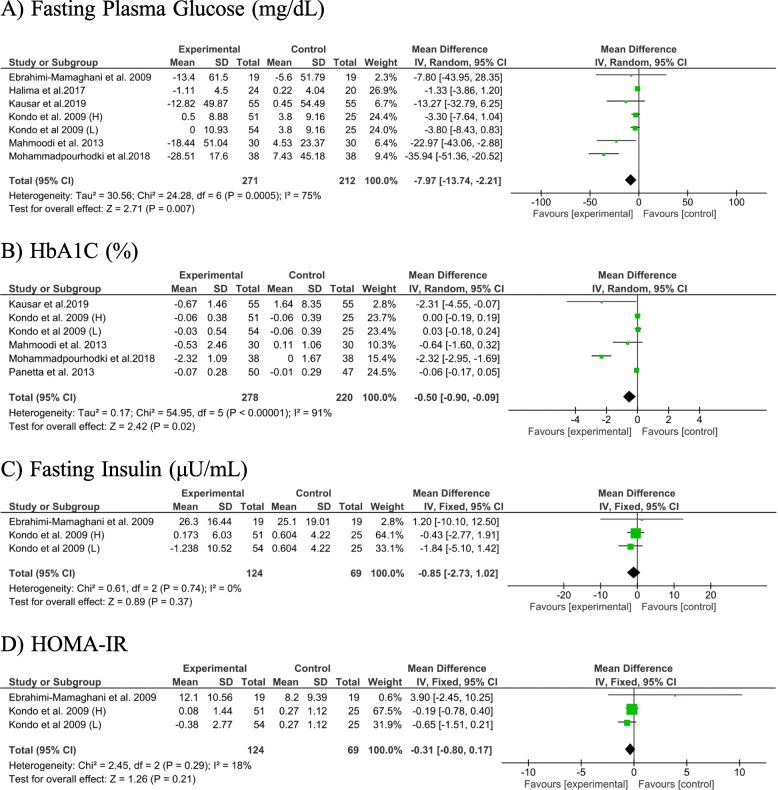
The meta-analysis results of the effect of apple cider vinegar administration on glycemic related factors. Kondo et al. study administrated apple cider vinegar in 2 different dosages which showed as “L” (lower dose) and “H” (higher dose) in figure

Pooled effect size from 6 studies, with 7 effect sizes, revealed a significant reduction in FPG (− 7.97 mg/dL; 95% CI: − 13.74, − 2.21, *P* = 0.007; *I*^*2*^: 75%) after ACV consumption. The same findings were obtained for HbA1C, when we combined 6 effect sizes from 5 studies (− 0.50 mg/dL; 95% CI: − 0.90, − 0.09, *P* = 0.02; *I*^*2*^: 91%). No significant effect of ACV consumption was found on serum insulin levels (− 0.85 mg/dL; 95% CI: − 2.73, 1.02, *P* = 0.37; *I*^*2*^: 0%) and HOMA-IR (− 0.31 mg/dL; 95% CI: − 0.80, 0.17, *P* = 0.21; *I*^*2*^: 18%) (Fig. 3).

Subgroup analysis revealed a significant reduction in FPG in studies recruited non-diabetics (− 3.53 mg/dL; 95% CI: − 6.70, − 0.37; *I*^*2*^: 0%). Such an effect was not observed in studies that enrolled diabetic patients (− 16.28 mg/dL; 95% CI: − 33.02, − 0.47; *I*^*2*^: 83%). Furthermore, when studies were stratified based on duration of intervention, the lowering effect of ACV on FPG was found in studies with an intervention of > 8-weeks follow-up (− 3.78 mg/dL; 95% CI: − 6.90, − 0.66; *I*^*2*^: 0%). No significant effect of ACV consumption on FPG was seen in studies with a low or high dose of ACV. When we excluded the study of Mohammadpourhodki et al. (36), between-study heterogeneity became non-significant (− 3.09 mg/dL; 95% CI: − 5.83, − 0.35; *I*^*2*^:23%). In addition, the lowering effect of ACV on HbA1C was non-significant in all subgroups (Table 4). Due to the low number of trials in each subgroup, stratified analysis was not conducted for serum insulin levels and HOMA-IR.

### Meta-regression

Findings from the meta-regression indicated an inverse association between change in FPG and HbA1C levels and baseline levels of these indicators (FPG: coefficient: -0.24; 95% CI: − 0.40, − 0.07; HbA1C: coefficient: -0.21; 95% CI: − 0.39, − 0.03). However, the effect of ACV intake on FPG and HbA1C was independent of the dose of ACV and duration of the study. Furthermore, no association was observed between change in other outcomes of interest following ACV intake and baseline measures, the dose of intervention, and duration of follow-up (Supplemental Table 2).

### Sensitivity analysis

Sensitivity analysis was performed by excluding individual studies from the meta-analysis. We found that our findings about serum TG was influenced by 3 studies, and when we removed each of these studies from the analysis, the findings did change [excluding Panetta et al. [33] WMD: − 39.38 mg/dL; 95% CI: − 73.18, − 5.59; excluding Mahmoodi et al. [27] WMD: − 39.46 mg/dL; 95% CI: − 75.37, − 3.55; and excluding Khezri et al. [22] WMD: − 20.76 mg/dL; 95% CI: − 36.86, − 4.65]. This was also the case when we removed the study of Bashiri et al. [4] in our analysis on serum HDL-C levels (WMD: 1.68 mg/dL; 95% CI: 0.44, 2.92); such that serum HDL-C levels were significantly increased by ACV consumption. With regards to HbA1C, we found that the study by Mohammadpourhodki et al. [28] had a significant effect on the overall finding; such that after removing that study from the analysis, no significant effect on HbA1C was seen following ACV consumption (− 0.04%; 95% CI -0.18, 0.10).

In addition, when we excluded studies with a high-risk of bias [12, 16, 27, 28], no alterations in findings occurred. Except for the findings of HbA1C, which became non-significant (− 0.03%; 95% CI: − 0.16, 0.10) (Supplemental Figure 1).

### Publication bias

Funnel plot showed a slight to moderate asymmetry in some variables (Supplemental Figure 2). Based on Egger’s regression asymmetry and Begg’s rank correlation test, we found no evidence of publication bias in studies on TG, TC, HDL-C, and HbA1C. However, a significant asymmetry was found in studies on serum LDL-C (*P* = 0.005) and FPG (*P* = 0.04) according to Egger’s regression test, although, such results were not confirmed by Begg’s rank-correlation test (LDL-C: *P* = 0.40; FPG: *P* = 0.17).

## Discussion

The present systematic review and meta-analysis suggested that ACV consumption yielded beneficial effects on serum TC and FPG levels. In addition, a trend toward a significant favorable effect was also observed in serum TG concentrations.

Hyperlipidemia and hyperglycemia are common metabolic disorders that affect many people around the world [34, 36]. In spite of several strategies to manage these abnormalities, lifestyle modifications are the first line of therapy in these conditions. Indeed, findings from the present meta-analysis highlight the application of ACV, as a dietary agent, may be helpful in controlling these metabolic abnormalities [9].

The present study showed that ACV consumption improved serum levels of FPG. With regard to HbA1C, despite the significant overall effect of ACV, we found that the exclusion of one study resulted in a non-significant finding, indicating that the overall findings were study-dependent. The mechanism of the ACV effect on lipid profiles and glycemic related markers has not been well defined; however, empirical studies have suggested several potential mechanisms. Indeed, ACV can improve glycemic status by delaying gastric emptying, enhancing cellular glucose utilization and lipolysis, suppressing hepatic glucose production and lipogenesis, and facilitating insulin secretion [21, 35]. Furthermore, in our study, the beneficial effect of ACV on FPG levels was more pronounced when the duration of studies lasted > 8 weeks. Subgroup analysis revealed that the FPG lowering effect of ACV was not significant in non-diabetic patients. On the other hand, the meta-regression results indicated a negative association between changes in both HbA1C and FPG levels, and their baseline measures. These findings suggest that higher baseline values of FPG and HbA1C might contribute to a greater reduction in these markers following ACV intake.

This study revealed that ACV consumption might reduce serum TC concentrations; where the effect of ACV on lipid profiles might be attributed to its stimulation of acid bile excretion, increasing lipolysis and decreasing lipogenesis [21, 35]. Subgroup analysis indicated a greater beneficial effect on both TC and TG levels among type 2 diabetics patients. In addition, the effect on TG and TC was more notable when interventions lasted > 8 weeks. The results from subgroup analysis also showed a greater lowering effect of ACV on TG and TC levels in doses of ≤15 ml/day. Therefore, 15 ml/day might represent the optimum effective dose of ACV. However, our findings from meta-regression analysis did not indicate an association between the dosage of ACV intake and serum alterations of these parameters. Nevertheless, the null results from meta-regression analysis might be due to the paucity of data in terms of ACV doses in published studies. We also found a significant improvement in HDL-C among non-diabetic participants. Given that diabetic patients are susceptible to higher levels of oxidative stress and the reductions of the expression and/or activity of HDL-C’s anti-oxidative enzymes, such as paraoxonase, in these patients, it is reasonable to expect a higher dosage of ACV would be more effective in these individuals. Therefore, further investigations would be required to shed light on this issue in diabetic patients.

ACV appears to be a safe natural supplement with a functional role in controlling glycemic and lipid profiles. Only two studies [4, 20] had reported some side effects (such as stomach burning and ACV intolerance) following consumption of this supplement. The present systematic review and meta-analysis has some limitations which must be taken into account. The number of included studies, in particular in terms of insulin and HOMA-IR, was relatively low, thereby precluding reliable conclusions to be drawn. Between-study heterogeneity was high for some outcomes, and although we endeavoured to find the source of heterogeneity in subgroup analysis, the heterogeneity remained significant in some subgroups. Most studies did not control the participant’s dietary intake, which might influence study outcomes. Finally, some trials were potentially high risk of bias in methodology, especially in blinding. However, except for HbA1C, the findings did not change by excluding studies with a high risk of bias.

## Conclusion

Following systematic review and meta-analysis, we found that ACV consumption might beneficially affect glycemic status and lipid parameters in adults; however, due to some limitations, the findings should be interpreted with caution. Considering that ACV is a safe food, it could be considered as a functional food and adjuvant therapy in the management of metabolic abnormalities. However, further studies are needed to clarify all possible beneficial effects of ACV on glycemic markers and lipid profiles.

## Supplementary Information

**Additional file 1: Supplemental Figure 1.** Sensitivity analysis. Abbreviations: TG: Triacylglycerol; TC: Total-Cholesterol; LDL-C: Low-density Lipoprotein Cholesterol; HDL-C: High-density Lipoprotein Cholesterol; FBS: Fasting Blood Glucose.

**Additional file 2: Supplemental Figure 2.** Funnel plot illustrating publication bias in the studies reporting effect of apple cider vinegar intake on the lipid profiles and glycemic related markers. Abbreviations: TG: Triacylglycerol; TC: Total-Cholesterol; LDL-C: Low-density Lipoprotein Cholesterol; HDL-C: High-density Lipoprotein Cholesterol; FBS: Fasting Blood Glucose; HOMA-IR: Homeostatic Model Assessment of Insulin Resistance.

**Additional file 3: Supplemental Table 1.** The search strategy used for each database.

**Additional file 4: Supplemental Table 2.** Meta-regression of the association between the change in outcomes of interest response to ACV intake and potential moderator.

## Data Availability

Not applicable.

## Abbreviations

CVDs: Cardiovascular diseases
ACV: Apple cider vinegar
PRISMA: Preferred Reporting Items for Systematic Reviews and Meta- Analysis
MeSH: Medical Subject Heading
MD: Mean differences
SDs: Standard deviations
FPG: Fasting plasma glucose
HOMA-IR: Homeostasis model assessment of insulin resistance
TG: Triacylglycerol
TC: Total cholesterol
LDL-C: Low-density lipoprotein cholesterol
HDL-C: High-density protein cholesterol
