# Supplementary material for: The effect of apple cider vinegar on lipid profiles and glycemic parameters: a systematic review and meta-analysis of randomized clinical trials
Source: BMC Complement Med Ther. 2021 Jun 29;21:179. doi: 10.1186/s12906-021-03351-w (PMC8243436; doi:10.1186/s12906-021-03351-w)
Supplement: Supplementary file 1 — Additional file 1: Supplemental Figure 1. Sensitivity analysis. Abbreviations: TG: Triacylglycerol; TC: Total-Cholesterol; LDL-C: Low-density Lipoprotein Cholesterol; HDL-C: High-density Lipoprotein Cholesterol; FBS: Fasting Blood Glucose. [file 12906_2021_3351_MOESM1_ESM.docx]

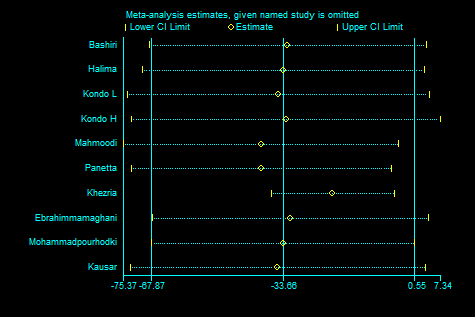

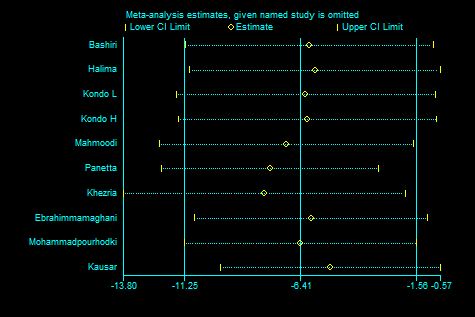


A) TG B) TC


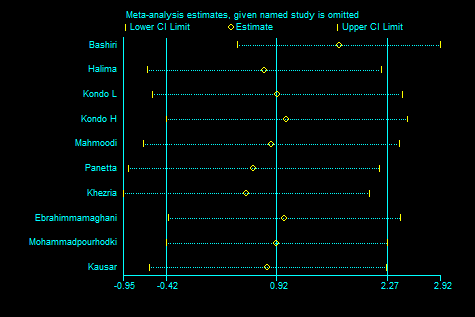

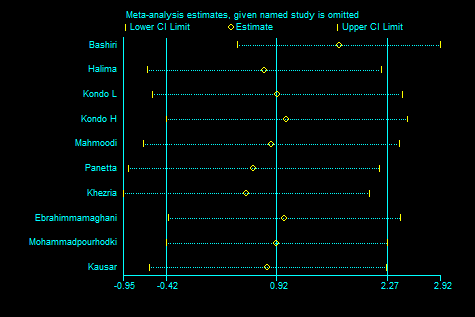


C) HDL-C D) LDL-C


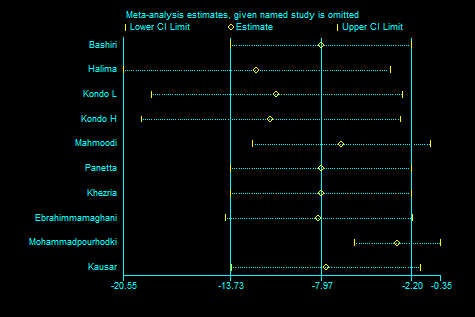

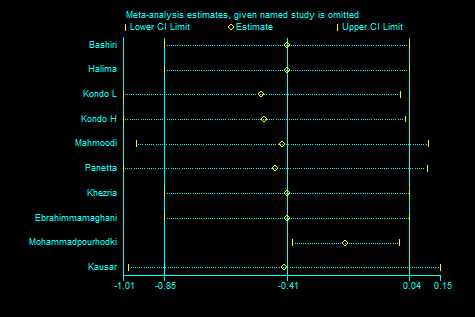


E) FBS F) HbA1C

**Supplemental Figure 1**. Sensitivity analysis. Abbreviations: TG: Triacylglycerol; TC: Total-Cholesterol; LDL-C: Low-density Lipoprotein Cholesterol; HDL-C: High-density Lipoprotein Cholesterol; FBS: Fasting Blood Glucose.
