# Supplementary material for: The effect of apple cider vinegar on lipid profiles and glycemic parameters: a systematic review and meta-analysis of randomized clinical trials
Source: BMC Complement Med Ther. 2021 Jun 29;21:179. doi: 10.1186/s12906-021-03351-w (PMC8243436; doi:10.1186/s12906-021-03351-w)
Supplement: Supplementary file 2 — Additional file 2: Supplemental Figure 2. Funnel plot illustrating publication bias in the studies reporting effect of apple cider vinegar intake on the lipid profiles and glycemic related markers. Abbreviations: TG: Triacylglycerol; TC: Total-Cholesterol; LDL-C: Low-density Lipoprotein Cholesterol; HDL-C: High-density Lipoprotein Cholesterol; FBS: Fasting Blood Glucose; HOMA-IR: Homeostatic Model Assessment of Insulin Resistance. [file 12906_2021_3351_MOESM2_ESM.docx]

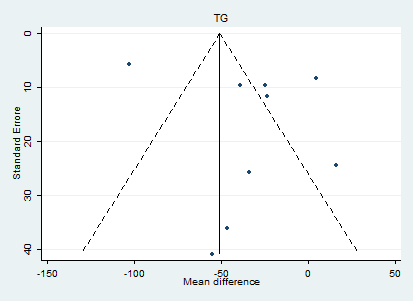

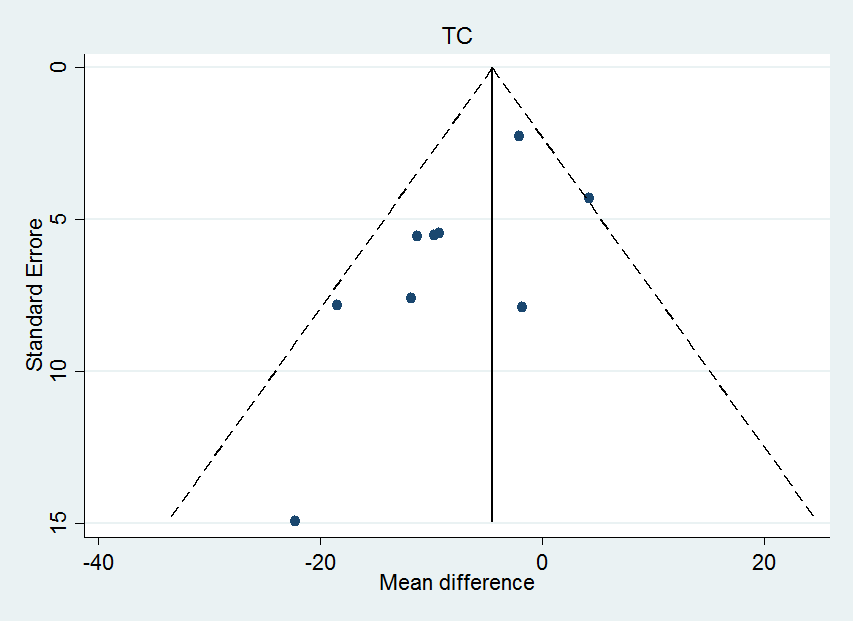

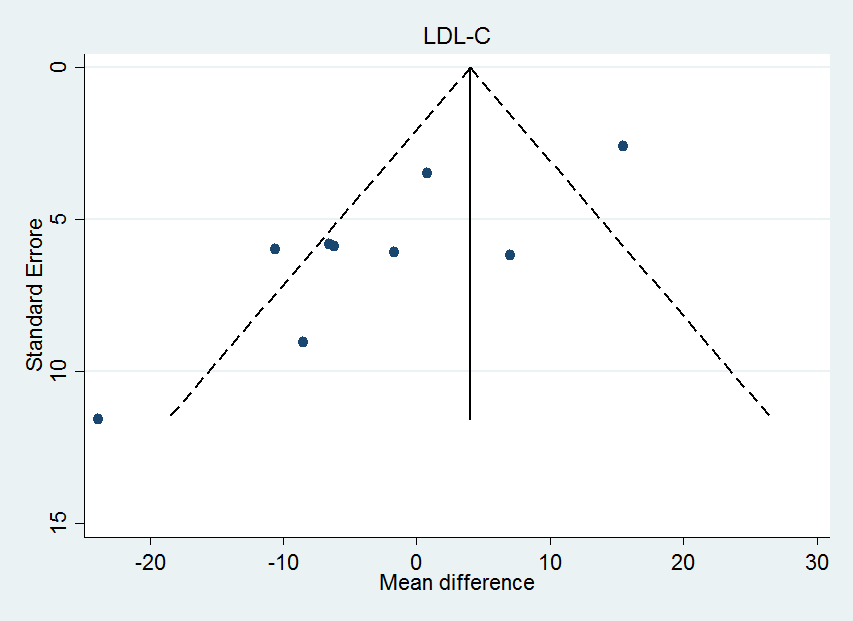

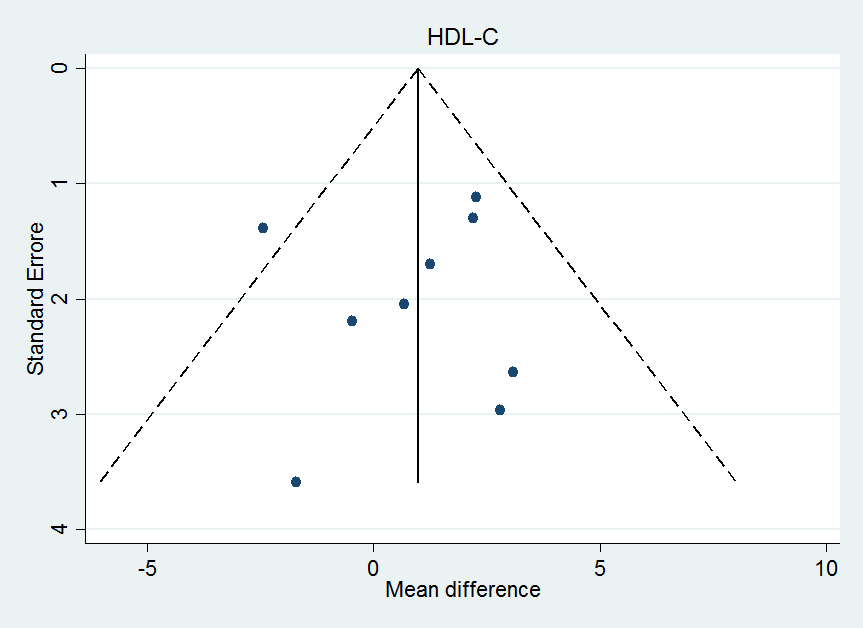

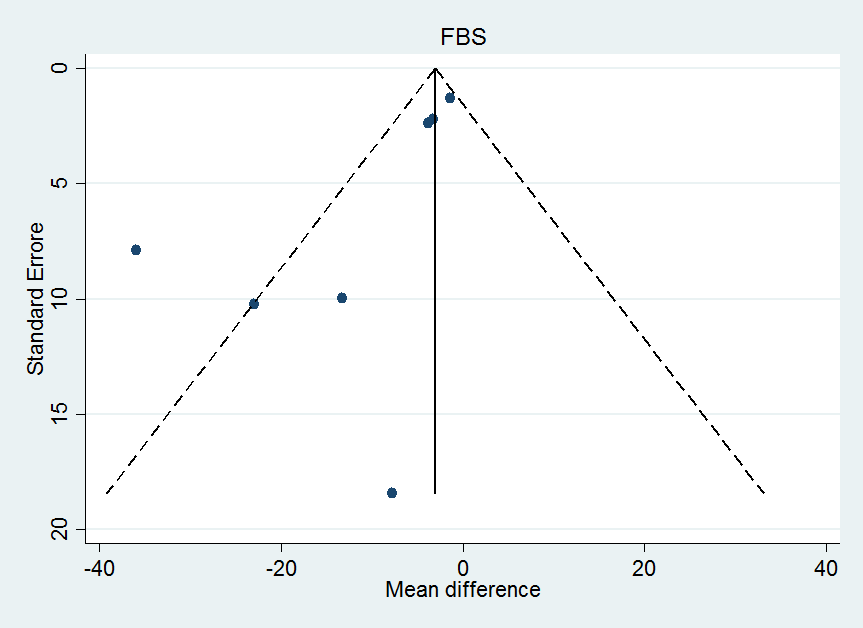

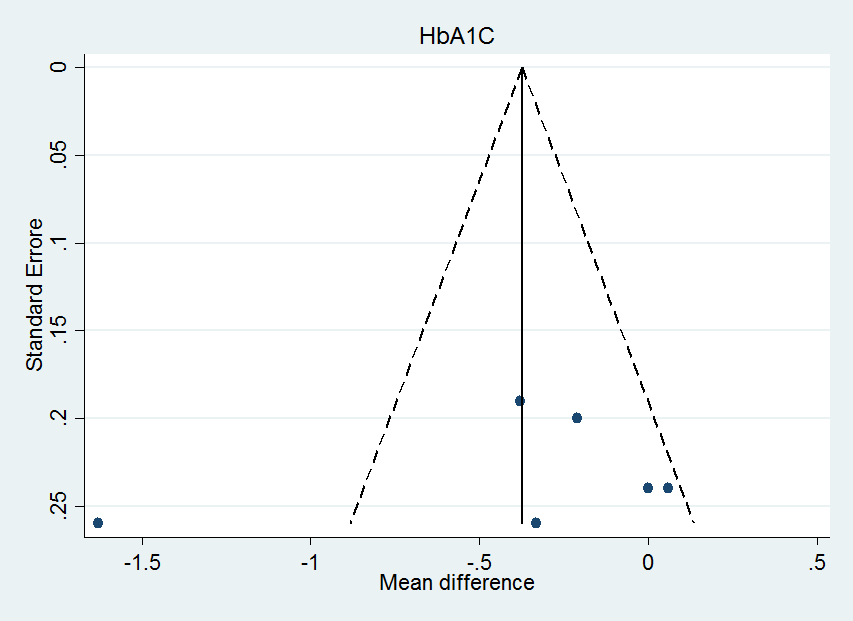

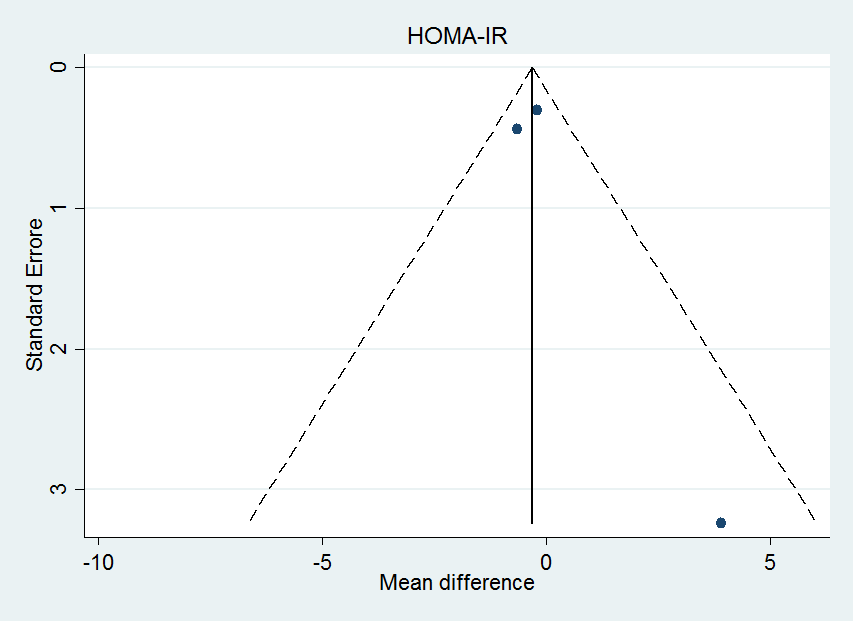

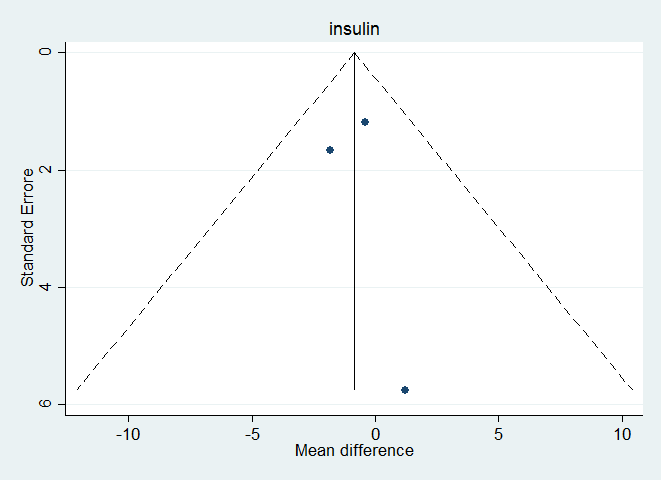


**Supplemental Figure 2**. Funnel plot illustrating publication bias in the studies reporting effect of apple cider vinegar intake on the lipid profiles and glycemic related markers. Abbreviations: TG: Triacylglycerol; TC: Total-Cholesterol; LDL-C: Low-density Lipoprotein Cholesterol; HDL-C: High-density Lipoprotein Cholesterol; FBS: Fasting Blood Glucose; HOMA-IR: Homeostatic Model Assessment of Insulin Resistance.
