# Supplementary material for: The effect of apple cider vinegar on lipid profiles and glycemic parameters: a systematic review and meta-analysis of randomized clinical trials
Source: BMC Complement Med Ther. 2021 Jun 29;21:179. doi: 10.1186/s12906-021-03351-w (PMC8243436; doi:10.1186/s12906-021-03351-w)
Supplement: Supplementary file 3 — Additional file 3: Supplemental Table 1. The search strategy used for each database. [file 12906_2021_3351_MOESM3_ESM.docx]

**Supplemental table 1.** The search strategy used for each database.

| Databases | Keywords | Results |
| --- | --- | --- |
| PubMed | ("Acetic Acid"[MeSH Terms] OR "vinegar"[Title/Abstract] OR "acetic acid"[Title/Abstract] OR "Apple cider vinegar"[Title/Abstract] OR "ACV"[Title/Abstract] OR "fermented apple"[Title/Abstract]) AND ("randomized controlled trial"[Publication Type] OR "controlled clinical trial"[Publication Type] OR "controlled clinical trial"[All Fields] OR randomized[Title/Abstract] OR randomised[Title/Abstract] OR placebo[Title/Abstract] OR "clinical trials as topic"[MeSH Terms] OR "cross-over studies"[MeSH Terms] OR "cross-over studies"[All Fields] OR "cross over studies"[All Fields] OR "Cross-over study"[All Fields] OR "Cross over study"[All Fields] OR "clinical trial"[Publication Type] NOT animals[All Fields]) | 998 |
| Scopus | ( TITLE-ABS-KEY ( ( ( "vinegar" ) OR ( "acetic acid" ) OR ( "Apple cider vinegar" ) OR ( "fermented apple" ) ) ) AND TITLE-ABS-KEY ( ( ( "randomized controlled trial" ) OR ( "controlled clinical trial" ) OR ( "randomized" ) OR ( "randomised" ) OR ( "placebo" ) OR ( "clinical trials as topic" ) OR ( "cross-over studies" ) OR ( "cross over studies" ) OR ( "Cross-over study" ) OR ( "Cross over study" ) OR ( "clinical trial" ) ) ) ) AND ( LIMIT-TO ( DOCTYPE , "ar" ) ) AND ( LIMIT-TO ( LANGUAGE , "English" ) ) | 3124 |
| Cochrane | (("vinegar") OR ("acetic acid") OR ("Apple cider vinegar") OR ("ACV") OR ("fermented apple")) in Title Abstract Keyword AND ( ( "randomized controlled trial" ) OR ( "controlled clinical trial" ) OR ( "randomized" ) OR ( "randomised" ) OR ( "placebo" ) OR ( "clinical trials as topic" ) OR ( "cross-over studies" ) OR ( "cross over studies" ) OR ( "Cross-over study" ) OR ( "Cross over study" ) OR ( "clinical trial" ) ) in Title Abstract Keyword | 1087 |
| Web of Science | TS=((("vinegar") OR ("acetic acid") OR ("Apple cider vinegar") OR ("ACV") OR ("fermented apple")) AND (("randomized controlled trial") OR ("controlled clinical trial") OR (“randomized”) OR (“randomized”) OR (“placebo”) OR ("clinical trials as topic") OR ("cross-over studies") OR ("cross over studies") OR ("Cross-over study”) OR ("Cross over study") OR ("clinical trial"))) | 802 |
