# Supplementary material for: The effect of apple cider vinegar on lipid profiles and glycemic parameters: a systematic review and meta-analysis of randomized clinical trials
Source: BMC Complement Med Ther. 2021 Jun 29;21:179. doi: 10.1186/s12906-021-03351-w (PMC8243436; doi:10.1186/s12906-021-03351-w)
Supplement: Supplementary file 4 — Additional file 4: Supplemental Table 2. Meta-regression of the association between the change in outcomes of interest response to ACV intake and potential moderator. [file 12906_2021_3351_MOESM4_ESM.docx]

**Supplemental table 2.** Meta-regression of the association between the change in outcomes of interest response to ACV intake and potential moderator.

| **Variables** | **Baseline measures** | **Duration** | **Amount of administration** |
| --- | --- | --- | --- |
| **TG** | -0.16 (-1.02, 0.70) | -0.71 (-2.07, 0.64) | -0.03 (-0.21, 0.14) |
| **TC** | 0.05 (-0.50, 0.60) | -0.04 (-0.34, 0.25) | -0.01 (-0.06, 0.03) |
| **LDL-C** | 0.48 (-0.12, 1.09) | -0.004 (-0.42, 0.41) | -0.03 (-0.07, 0.01) |
| **HDL-C** | -0.04 (-0.26, 0.18) | 0.004 (-0.08, 0.09) | -0.003 (-0.01, 0.008) |
| **FBS** | **-0.24 (-0.40, -0.07)** | 0.09 (-0.48, 0.68) | 0.004 (-0.07, 0.08) |
| **HbA1C** | **-0.21 (-0.39, -0.03)** | 0.008 (-0.02, 0.04) | 0.01 (-0.10, 0.12) |

Abbreviations: TG: Triacylglycerol; TC: Total-Cholesterol; LDL-C: Low-density Lipoprotein Cholesterol; HDL-C: High-density Lipoprotein Cholesterol; FBS: Fasting Blood Glucose; HOMA-IR: Homeostatic Model Assessment of Insulin Resistance.
